# Juvenile‐onset and adult‐onset demodicosis in dogs in the UK: prevalence and breed associations

**DOI:** 10.1111/jsap.13067

**Published:** 2019-10-04

**Authors:** D. G. O'Neill, E. Turgoose, D. B. Church, D. C. Brodbelt, A. Hendricks

**Affiliations:** ^1^ Production and Population Health The Royal Veterinary College Hatfield Herts AL9 7TA UK; ^2^ Clinical Sciences and Services The Royal Veterinary College Hatfield Herts AL9 7TA UK

## Abstract

**Objectives:**

To explore epidemiological features of demodicosis relevant to UK veterinary general practitioners. Breed risk factors were proposed as distinct between juvenile‐onset and adult‐onset disease.

**Materials and Methods:**

The study used anonymised clinical data on dogs under primary veterinary care at practices enrolled in the UK VetCompass Programme. Case inclusion required recording of a final demodicosis diagnosis for a dermatological condition that was present during the 2013 study period. Risk factor analysis used multivariable logistic regression modelling.

**Results:**

In dogs aged <2 years (juvenile‐onset), the 1‐year period prevalence was 0.48% (95% confidence interval: 0.45 to 0.52). Compared with crossbred dogs, seven breeds showed increased odds of diagnosis with demodex: British bulldog, Staffordshire bull terrier, Chinese shar‐pei, dogue de Bordeaux, pug, French bulldog and boxer. Additionally, six breeds showed reduced odds of juvenile demodicosis: Lhasa apso, bichon frise, Labrador retriever, German shepherd dog, shih‐tzu and Chihuahua. In dogs aged >4 years (adult‐onset), the 1‐year period prevalence was 0.05% (95% confidence interval: 0.0.04 to 0.06). Six breeds showed increased odds of demodicosis compared with crossbred dogs: Chinese shar‐pei, shih‐tzu, West Highland white terrier, pug, boxer and Border terrier.

**Clinical Significance:**

Juvenile‐onset demodicosis is much more common (about 10 times higher) than the adult‐onset form. Knowledge of the predisposed breeds for these two presentations can assist with diagnosis and support the concept of distinct aetiopathogenetic phenotypes.

## INTRODUCTION

Demodicosis is a relatively common skin disease in dogs that occurs when the normally harmless and commensal Demodex spp. mites in hair follicles and/or sebaceous glands multiply to excessive numbers (Miller *et al*. 2013). Two species of demodectic mites are associated with disease in dogs. The most frequently recognised of these, *Demodex canis*, was first recognised in 1859 (Leydig 1859) and there is also *Demodex injai* (Hillier & Desch 2002, Sastre *et al*. 2012, Mueller *et al*. 2018). The clinical presentation of demodicosis shows wide variation in the age at onset, the extent and severity of the lesions and the presence of secondary infection, in addition to the mite species involved (Ordeix *et al*. 2009, Miller *et al*. 2013). Demodicosis is often associated with secondary bacterial skin infection (Kuznetsova *et al*. 2012), and deep and extensive pyoderma can lead to serious, debilitating or even life‐threatening morbidity in generalised disease (Miller *et al*. 2013). Due to the suspected genetic factors involved, breeding from affected dogs is often discouraged and greater understanding of breed‐associations is needed (Scott *et al*. 2001).

Canine demodicosis is commonly classified by either the age at onset (juvenile *versus* adult) and/or the extent of the disease (localised *versus* generalised) to assist with prognostic advice and to guide diagnostic and management approaches (Mueller *et al*. 2012). Given that very low numbers of *D. canis* inhabit haired skin of dogs without skin disease (Ravera *et al*. 2013), the question arises as to why some of these dogs develop skin lesions from which large numbers of mites can be recovered. In some affected dogs, lesions with abnormally high numbers of mites are few and transient (mild localised disease), while others show large areas and/or multiple sites affected by lesions that do not resolve without treatment (generalised disease) (Scott *et al*. 2001). Although genetic or acquired immunosuppression affecting control of mite populations is generally assumed to be central to the pathogenesis (Gross *et al*. 2005b, Miller *et al*. 2013), the genetics, immunology and pathogenesis of demodicosis remain poorly understood and are likely to be highly complex (Ferrer *et al*. 2014).

Many dogs with resolved juvenile‐onset demodicosis do not have recurrent disease in later adulthood (Bowden *et al*. 2018). Many dogs with adult‐onset disease had previously had seemingly controlled mite populations for years (Miller *et al*. 2013). Many dogs that sustain immunosuppression through disease or treatment as either juveniles or adults never develop demodicosis (Miller *et al*. 2013). Given these outcomes, distinct risk factors are likely to exist between juvenile‐onset and adult‐onset demodicosis that may offer routes to better understanding and management of the condition.

Reported prevalence values for canine demodicosis vary widely, from 0.4 to 23% (Sischo *et al*. 1989, Scott & Paradis 1990, Nayak *et al*. 1997, Rodriguez‐Vivas *et al*. 2003, Chee *et al*. 2008, Plant *et al*. 2011, Bowden *et al*. 2018). This wide prevalence range may result from studies using skewed populations from diverse geographical locations and that may not distinguish between the different forms of the disease. Multiple, and often conflicting, risk factors and breed predispositions have been proposed and re‐cited widely, although the strength of evidence for many of these original studies is often questionable (Scott & Paradis 1990, Lemarié *et al*. 1996, Nayak *et al*. 1997, Gross *et al*. 2005a, Gortel 2006, Mueller *et al*. 2009, Schnabl *et al*. 2010, Plant *et al*. 2011, Kuznetsova *et al*. 2012, Mueller 2012, Mueller *et al*. 2012, Miller *et al*. 2013, Bowden *et al*. 2018). Some larger and more recent studies from the USA reported prevalence values of 0.58% based on a diagnosis of juvenile demodicosis alone from the electronic records of a large network of veterinary practices (Plant *et al*. 2011) and 0.37% for demodicosis overall based on data from one teaching hospital (Bowden *et al*. 2018). Reports on breed as a risk factor or of breed representation differed markedly within these study groups, with bull dog and bull terrier types and shar‐pei dogs having high odds of diagnosis with juvenile demodicosis (Plant *et al*. 2011), but crossbred dogs were the most frequently represented dogs in the study of overall demodicosis (Bowden *et al*. 2018). The need for accurate and generalisable prevalence risk factor data on diseases with an inherited component has been highlighted as a key criterion for longer term reduction in disease and improvement in dog welfare (Bateson 2010).

The present study aimed to explore epidemiological features of canine demodicosis as relevant to veterinary practitioners in the UK, based on the general population of dogs under primary veterinary care practices enrolled in the UK VetCompass Programme. The specific objectives were: (1) to report the 1‐year period prevalence and age distribution of demodicosis in dogs of all ages attending primary care practices in the UK; and, (2) to report the 1‐year period prevalence and evaluate risk factors for demodicosis separately for dogs under 2 years of age (juvenile‐onset) and for those aged over 4 years (presumed adult‐onset). The study proposed that breed risk factors are distinct between the juvenile and adult‐onset forms of the disease.

## METHODS

The VetCompass Programme collates de‐identified electronic patient record (EPR) data from primary‐care veterinary practices in the UK for epidemiological research (O'Neill *et al*. 2014). VetCompass collects information fields that include species, breed, date of birth, sex, neuter status and bodyweight and clinical information from free‐form text clinical notes plus treatment with relevant dates. The EPR data were extracted from practice management systems using integrated clinical queries and uploaded to a secure VetCompass structured query language database (O'Neill *et al*. 2015).

The study used a cross‐sectional analysis based on cohort clinical data of dogs attending VetCompass practices (Dohoo *et al*. 2009, Pearce 2012). The sampling frame for the current study included dogs under veterinary care within the VetCompass database for a 1‐year period from January 1, 2013 to December 31, 2013. “Under veterinary care” was defined as requiring either: (1) at least one EPR recorded from January 1 to December 31, 2013; or, (2) at least one EPR both before and after 2013. Sample size calculations estimated that at least 60,752 dogs would need to be sampled to estimate a disorder that had 0.4% prevalence with 0.05% acceptable margin of absolute error at a 95% confidence level assuming a UK population of 8 million dogs (Asher *et al*. 2011, Epi Info 7 CDC 2019). Ethical approval was granted by the RVC Ethics and Welfare Committee (reference number 2016/U37).

Case inclusion criteria required that a final diagnosis of demodicosis was recorded in the EPR for a dermatological condition during the 2013 study period. These final diagnoses relied on the clinical acumen of the veterinary teams providing clinical care to the study population over time. No additional specific inclusion or exclusion criteria for diagnosis because the study aimed to identify all diagnosed cases in the primary‐care setting. These cases could have been first diagnosed during 2013 (incident cases) or ongoing cases first diagnosed before 2013 (pre‐existing cases). Case‐finding involved initial screening of all 455,553 study dogs for candidate demodicosis cases by searching the clinical free‐text field using the search term “demod*” with fuzziness to allow two‐letter inversion, insertion or deletion (Paiva 2013). Candidate cases were randomly ordered and the clinical notes of all 3307 candidate animals were manually reviewed in detail to evaluate them for case inclusion.

Breed information recorded in the clinical records was mapped to a standardised breed list adapted from the VeNom Coding system (The VeNom Coding Group 2019). A *breed* variable included individual breeds represented by >5000 dogs and/or with ≥10 demodicosis cases in the overall study, a general grouping of crossbred dogs (*i.e*. non‐purebred dogs) and a grouped category of all remaining dogs. This approach was taken to facilitate statistical power for the individual breed analyses (Scott *et al*. 2012). A *purebred* variable categorised all dogs of recognisable breeds as “purebred” and the remaining dogs as “crossbred” (Irion *et al*. 2003). Sex‐neuter (female‐entire, female‐neutered, male‐entire, male‐neutered, unrecorded) described the status recorded at the final EPR. Age described the age (years) at first diagnosis for incident demodicosis cases during 2013 and age at the final date of the study period for non‐cases (December 31, 2013) by which point these dogs had not become cases. An *age* variable categorised age into six groups (<1.0, 1.0 to <2.0, 2.0 to 4.0, >4 to <7.0, 7.0 to <10.0, ≥10.0). Adult bodyweight described the maximum bodyweight (kg) recorded for dogs >18 months at any time in the available clinical records. An *adult bodyweight* variable categorised adult bodyweight into six groups (<10.0, 10.0 to 19.9, 20.0 to 29.9, 30.0 to 39.9, ≥40.0, unavailable). A *bodyweight relative to breed/sex mean* variable characterised the adult bodyweight of individual dogs as either below or equal/above the mean adult bodyweight for their breed type and sex within the overall study population. Crossbred was included as a single breed type for this variable. This variable allowed the effect of adult bodyweight to be assessed *within* each breed/sex combination.

Following data checking and cleaning in Excel (Microsoft Office Excel 2013; Microsoft Corp.), analyses were conducted using Stata Version 13 (Stata Corporation). Period prevalence refers to the number of cases that are known to have existed at any time during a specified period (*e.g*. 1 year) and is generally reported as the percentage of cases from all animals that were at risk. Prevalence describes all cases, whether incident or pre‐existing (Thrusfield 2007). One‐year period prevalence with 95% confidence intervals (CIs) described the probability of being a demodicosis case at any time during the 1‐year 2013 study period and was reported for the study dogs overall, for dogs aged <2 years and for dogs aged >4 years and also for individual breeds. Binomial CI estimates were derived using the Clopper–Pearson exact method (Brown *et al*. 2001). Risk factor analysis for demographic associations with demodicosis was carried out separately for dogs aged <2 years and for dogs aged >4 years. Binary logistic regression modelling was used to evaluate unconditional associations between hypothesised risk factors (*breed, purebred, adult bodyweight, bodyweight relative to breed/sex mean, age category, sex, neuter*) and demodicosis during 2013. Based on review of the current literature on reported risk factors and pathogenesis of demodicosis within possible causal pathways (Shrier & Platt 2008), the following factors were allowable for inclusion in multivariable evaluation to build a breed‐multivariable model: *breed, bodyweight relative to breed/sex mean, age, sex‐neuter*. Because breed was a factor of primary interest for the study, *purebred* (derived from the breed variable) and a*dult bodyweight* (a defining characteristic of individual breeds) were not considered in the same multivariable modelling. Instead, *purebred* replaced the *breed* variable in the final breed‐multivariable while a*dult bodyweight* replaced the *breed* and *bodyweight relative to breed/sex mean* variables. Model development used manual backwards stepwise elimination. The likelihood ratio test was used to compare a random effects model with clinic entered as a random effect against the non‐random effects model with P<0.05 cut‐off used for selection of the random effects model. Pair‐wise interaction effects were evaluated using the likelihood ratio test for all variables retained in the final models, with P<0.05 cut‐off used for inclusion of biologically plausible interaction terms (Dohoo *et al*. 2009). Pearson and deviance residuals were checked and implausible outliers were either corrected or removed (Dohoo *et al*. 2009). The area under the ROC curve and the Hosmer‐Lemeshow test were used to evaluate the quality of the model fit and discrimination (non‐random effect model) (Dohoo *et al*. 2009, Hosmer *et al*. 2013).

## RESULTS

### Prevalence

**Figure 1:**
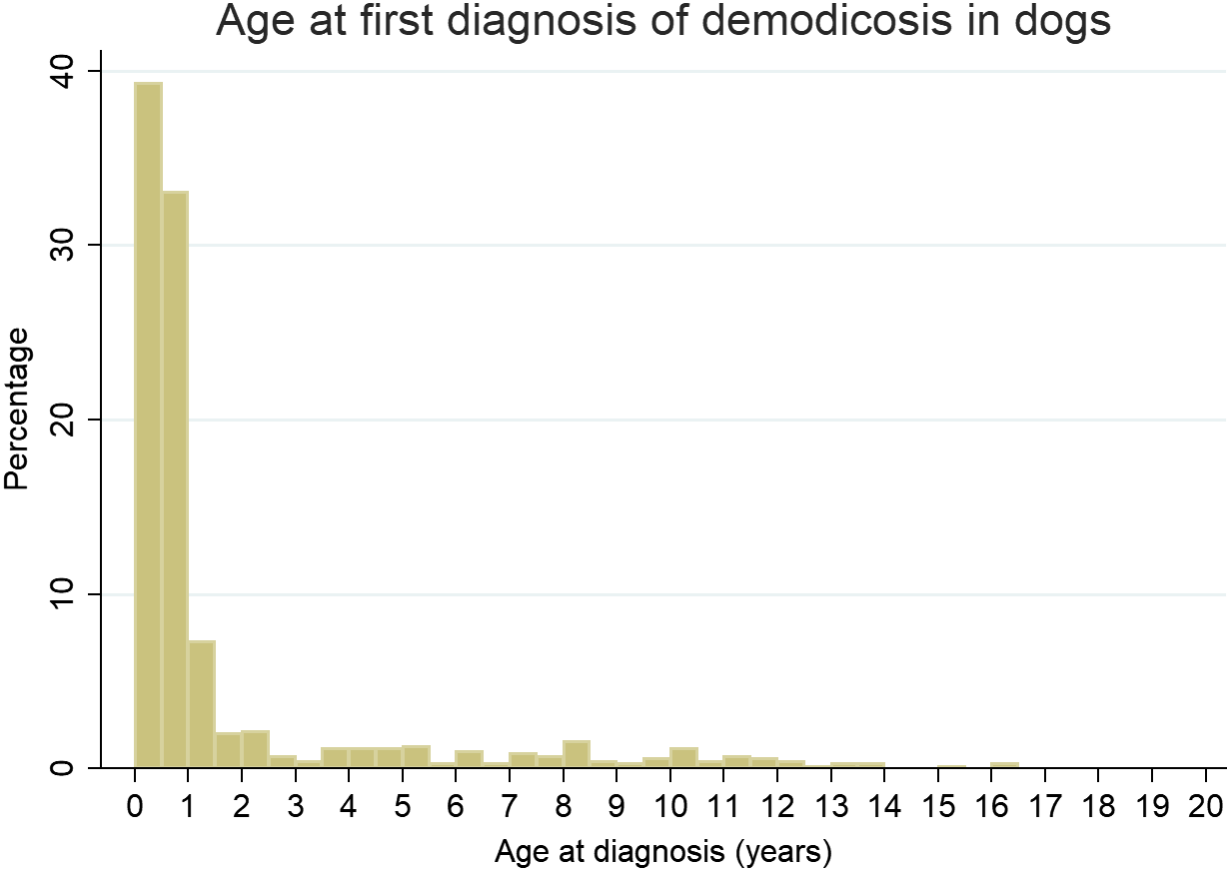
Age distribution of dogs under primary veterinary care in the UK at first diagnosis with demodicosis (n=702)

From dogs of all ages, there were 788 demodicosis cases confirmed from 455,553 dogs under veterinary care during 2013 at 304 clinics to give a 1‐year period prevalence for demodicosis overall of 0.17% (95% CI: 0.16 to 0.19). The breeds with the highest demodicosis 1‐year period prevalence across all ages were British bulldog, French bulldog, pug, dogue de Bordeaux and Chinese shar‐pei (Table 1). The age at first diagnosis was available for 702/788 (89.1%) cases. Of these 702 cases, the median age at first diagnosis overall was 7 months [interquartile range (IQR) 4 months to 13 months, range 1 month to 16 years and 2 months] (Fig. 1) and 508 (72.4%) were <1 year, 559 (79.6%) were <1.5 year, 573 (81.6%) were <2 years and 98 (14.0%) were >4 years.

**Table 1:** One‐year (2013) period prevalence of demodicosis for common and commonly‐affected dog breeds under primary veterinary care in the UK

	<2 years				>4 years				All ages			
Breed	Breed count (%)	No. (%) cases	Breed prevalence %	95% CI	Breed count (%)	No. (%) cases	Breed prevalence %	95% CI	Breed count (%)	No. (%) cases	Breed prevalence %	95% CI
British bulldog	1274 (1.0)	47 (7.7)	3.56	2.72 to 4.88	1149 (0.5)	1 (0.9)	0.09	0.00 to 0.48	3374 (0.7)	51 (6.5)	1.51	1.13 to 1.98
Staffordshire bull terrier	8.746 (7.0)	191 (31.2)	2.14	1.89 to 2.51	16,306 (7.1)	5 (4.3)	0.03	0.01 to 0.07	32,635 (7.2)	214 (27.2)	0.66	0.57 to 0.75
Pug	2566 (2.0)	50 (8.2)	1.91	1.44 to 2.56	1362 (0.6)	2 (1.7)	0.15	0.02 to 0.53	5376 (1.2)	53 (6.7)	0.99	0.74 to 1.29
French bulldog	1564 (1.2)	30 (4.9)	1.88	1.30 to 2.73	295 (0.1)	0 (0.0)	0.00	0.00 to 1.24	2397 (0.5)	31 (3.9)	1.29	0.88 to 1.83
Chinese shar‐pei	750 (0.6)	14 (2.3)	1.83	1.02 to 3.11	689 (0.3)	4 (3.4)	0.58	0.16 to 1.48	2034 (0.5)	18 (2.3)	0.88	0.53 to 1.40
Dogue de Bordeaux	720 (0.6)	13 (2.1)	1.77	0.96 to 3.07	589 (0.3)	1 (0.9)	0.17	0.00 to 0.94	1858 (0.4)	17 (2.2)	0.91	0.53 to 1.46
Boxer	1261 (1.0)	9 (1.5)	0.71	0.33 to 1.35	3794 (1.7)	4 (3.4)	0.11	0.03 to 0.27	6288 (1.4)	14 (1.8)	0.22	0.12 to 0.37
Border terrier	1215 (1.0)	5 (0.8)	0.41	0.13 to 0.96	3069 (1.3)	3 (2.6)	0.10	0.02 to 0.29	5449 (1.2)	8 (1.0)	0.15	0.06 to 0.29
Crossbreed	35,460 (28.2)	132 (21.5)	0.37	0.31 to 0.44	51,874 (22.7)	14 (12.0)	0.03	0.01 to 0.05	112,777 (24.8)	157 (19.9)	0.14	0.12 to 0.16
Rottweiler	1346 (1.1)	5 (0.8)	0.37	0.12 to 0.87	2916 (1.3)	2 (1.7)	0.07	0.01 to 0.25	5321 (1.2)	7 (0.9)	0.13	0.05 to 0.27
Cocker spaniel	4239 (3.4)	13 (2.1)	0.31	0.16 to 0.52	8053 (3.5)	2 (1.7)	0.02	0.00 to 0.09	15,827 (3.5)	18 (2.3)	0.11	0.07 to 0.18
Border collie	2708 (2.2)	7 (1.1)	0.26	0.10 to 0.53	7195 (3.2)	0 (0.0)	0.00	0.00 to 0.05	12,268 (2.7)	7 (0.9)	0.06	0.02 to 0.12
Others	22,883 (18.2)	48 (7.8)	0.21	0.15 to 0.28	44,357 (19.4)	16 (13.7)	0.04	0.02 to 0.06	86,548 (19.0)	74 (9.4)	0.09	0.07 to 0.11
Springer spaniel	1577 (1.3)	3 (0.5)	0.19	0.04 to 0.55	3444 (1.5)	1 (0.9)	0.03	0.00 to 0.16	6346 (1.4)	4 (0.5)	0.06	0.02 to 0.16
Jack Russell terrier	5720 (4.6)	10 (1.6)	0.17	0.08 to 0.32	16,208 (7.1)	2 (1.7)	0.01	0.00 to 0.05	27,691 (6.1)	12 (1.5)	0.04	0.02 to 0.08
Chihuahua	5716 (4.5)	9 (1.5)	0.16	0.07 to 0.30	2839 (1.2)	0 (0.0)	0.00	0.00 to 0.13	11,782 (2.6)	9 (1.1)	0.08	0.03 to 0.14
Shih tzu	5526 (4.4)	9 (1.5)	0.16	0.07 to 0.31	5561 (2.4)	23 (19.7)	0.41	0.26 to 0.62	15,038 (3.3)	34 (4.3)	0.23	0.16 to 0.32
German shepherd dog	2947 (2.3)	4 (0.7)	0.14	0.04 to 0.35	6966 (3.1)	5 (4.3)	0.07	0.02 to 0.17	12,520 (2.8)	9 (1.1)	0.07	0.03 to 0.14
Labrador retriever	7375 (5.9)	9 (1.5)	0.12	0.06 to 0.23	19,091 (8.4)	4 (3.4)	0.02	0.00 to 0.05	33,321 (7.3)	15 (1.9)	0.05	0.02 to 0.07
Cavalier King Charles spaniel	2075 (1.7)	2 (0.3)	0.10	0.01 to 0.35	5876 (2.6)	2 (1.7)	0.03	0.00 to 0.12	10,143 (2.2)	5 (0.6)	0.05	0.03 to 0.07
West Highland white Terrier	1709 (1.4)	1 (0.2)	0.06	0.00 to 0.33	8485 (3.7)	19 (16.2)	0.22	0.13 to 0.35	12,017 (2.6)	20 (2.5)	0.17	0.10 to 0.26
Lhasa apso	1969 (1.6)	1 (0.2)	0.05	0.00 to 0.28	3222 (1.4)	1 (0.9)	0.03	0.00 to 0.17	6840 (1.5)	2 (0.3)	0.03	0.00 to 0.11
Bichon	2035 (1.6)	1 (0.2)	0.05	0.00 to 0.27	2825 (1.2)	0 (0.0)	0.00	0.00 to 0.13	6607 (1.5)	1 (0.1)	0.02	0.00 to 0.08
Golden retriever	994 (0.8)	0 (0.0)	0.00	0.00 to 0.37	3752 (1.6)	3 (2.6)	0.08	0.02 to 0.23	5670 (1.2)	4 (0.5)	0.07	0.02 to 0.18
Yorkshire terrier	3435 (2.7)	0 (0.0)	0.00	0.00 to 0.11	8767 (3.8)	3 (2.6)	0.03	0.01 to 0.10	15,426 (3.4)	4 (0.5)	0.03	0.01 to 0.07

CI Confidence interval

Results are shown for all ages, dogs aged under 2 years and dogs aged over 4 years

From dogs aged <2 years, there were 613 demodicosis cases confirmed from 126,423 dogs to yield a 1‐year period prevalence of 0.48% (95% CI: 0.45 to 0.52). The breeds with the highest demodicosis 1‐year period prevalence among dogs aged <2 years were British bulldog, Staffordshire bull terrier, pug, French bulldog, Chinese shar‐pei and dogue de Bordeaux (Table 1). Of the dogs <2 years of age at diagnosis with complete data available for that variable, 301 (49.3%) cases were female compared with 59,386 (47.6%) of non‐cases and 203 (38.2%) of cases were neutered compared with 36,967 (33.7%) of non‐cases. There were 481 (78.5%) purebred cases compared with 89,858 (71.7%) purebred non‐cases. The median adult bodyweight of cases overall was 19.5 kg (IQR: 13.0 to 25.2) compared with 12.6 kg (IQR 7.6 to 24.0) for non‐cases.

From dogs aged >4 years, there were 117 demodicosis cases confirmed from 228,801 dogs to yield a 1‐year period prevalence of 0.05% (95% CI: 0.04 to 0.06). The breeds with the highest demodicosis 1‐year period prevalence among dogs aged >4 years were Chinese shar‐pei, shih‐tzu, West Highland white terrier, dogue de Bordeaux and pug (Table 1). Of the dogs >4 years of age with complete data available for that variable, 49 (41.9%) cases were female compared with 110,752 (48.5%) of non‐cases and 58 (63.0%) of cases were neutered compared with 122,028 (65.6%) of non‐cases. There were 103 (88.0%) purebred cases compared with 176,146 (77.3%) purebred non‐cases. The median adult bodyweight of cases was 11.8 kg (IQR: 8.8 to 25.0, range 4.1 to 58.8) compared with 18.3 kg (IQR 9.8 to 29.5, range 0.3 to 148.0) for non‐cases.

### Risk factors: Dogs under 2 years old

Six risk factors had univariable association with demodicosis at P<0.20 and were considered in multivariable modelling: *breed, purebred, sex‐neuter, age, adult bodyweight, bodyweight relative to breed/sex mean*. The final breed‐multivariable model comprised four risk factors: *breed, bodyweight relative to breed/sex mean, age and sex‐neuter*. The random effects model with clinic entered as a random effect was a better model of the data that the non‐random effects model (P<0.001). The intraclass correlation coefficient indicated that 5.9% of the unaccounted‐for variation in the data was due to unmeasured factors operating at the veterinary clinic level. No biologically plausible interactions at P<0.05 were detected. The final unclustered model showed acceptable model‐fit (Hosmer‐Lemeshow test statistic: P=0.311) and good discrimination (area under the ROC curve: 0.841). The frequencies of missing data for the 126,423 dogs in the final model variable were: *breed* n=492; 0.4%, *bodyweight relative to breed/sex mean* n=75,878; 60.0%, *age* n=0; 0.0% and *sex‐neuter* n=16,901; 13.4%.

After accounting for the effects of the other variables evaluated, seven breeds showed increased odds of demodicosis in dogs aged <2 years compared with crossbred dogs: British bulldog [odds ratio (OR): 11.26, 95% CI 7.94 to 15.97, P<0.001], Staffordshire bull terrier (OR: 7.11, 95% CI 5.65 to 8.93, P<0.001), Chinese shar‐pei (OR: 6.57, 95% CI 3.73 to 11.60, P<0.001), dogue de Bordeaux (OR: 5.92, 95% CI 3.30 to 10.62, P<0.001), pug (OR: 5.41, 95% CI 3.87 to 7.55, P<0.001), French bulldog (OR: 5.07, 95% CI 3.37 to 7.63, P<0.001) and boxer (OR: 2.04, 95% CI 1.03 to 4.04, P=0.040). There were six breeds with reduced odds of demodicosis compared with crossbreds: Lhasa apso, bichon frise, Labrador retriever, German shepherd dog, shih‐tzu and Chihuahua. Additionally, zero cases were identified for Yorkshire terrier and golden retriever. Dogs with an adult bodyweight that was equal or higher than their breed mean had 1.50 (95% CI 1.18 to 1.91, P=0.001) times the odds of demodicosis compared with dogs that weighed below their breed mean. Dogs aged 1.0 to <2.0 years had 0.12 (95% CI 0.09 to 0.15, P<0.001) times the odds of demodicosis compared with dogs aged <1.0 years. Compared with entire females, there were higher odds in neutered females (OR 1.44, 95% CI 1.10 to 1.90, P=0.008) and neutered males (OR 1.32, 95% CI 1.01 to 1.71, P=0.039) (Table 2).

**Table 2:** Final mixed‐effects breed‐multivariable logistic regression results for risk factors associated with diagnosis of demodicosis in dogs aged <2 years under primary veterinary care in the UK

Variable	Category	Cases	Total	Odds ratio	95% CI	Category P‐value	Variable P‐value
Breed	Crossbreed	132	35,592	Base			<0.001
	British bulldog	47	1321	11.26	7.94 to 15.97	**<0.001**	
	Staffordshire bull terrier	191	8937	7.11	5.65 to 8.93	**<0.001**	
	Chinese shar‐pei	14	764	6.57	3.73 to 11.60	**<0.001**	
	Dogue de Bordeaux	13	733	5.92	3.30 to 10.62	**<0.001**	
	Pug	50	2616	5.41	3.87 to 7.55	**<0.001**	
	French bulldog	30	1594	5.07	3.37 to 7.63	**<0.001**	
	Boxer	9	1270	2.04	1.03 to 4.04	**0.040**	
	Rottweiler	5	1351	1.16	0.47 to 2.85	0.743	
	Border terrier	5	1220	1.10	0.45 to 2.70	0.838	
	Cocker spaniel	13	4252	0.78	0.44 to 1.38	0.394	
	Border collie	7	2715	0.70	0.32 to 1.50	0.354	
	Others	48	22,931	0.60	0.43 to 0.84	**0.046**	
	Jack Russell terrier	10	5730	0.53	0.28 to 1.00	0.050	
	Springer spaniel	3	1580	0.51	0.16 to 1.61	0.254	
	Chihuahua	9	5725	0.48	0.24 to 0.94	**0.032**	
	Shih Tzu	9	5535	0.44	0.22 to 0.87	**0.018**	
	German shepherd dog	4	2951	0.42	0.15 to 1.13	**0.018**	
	Labrador retriever	9	7384	0.32	0.16 to 0.64	**0.001**	
	Cavalier King Charles spaniel	2	2077	0.26	0.07 to 1.07	0.062	
	West Highland White terrier	1	1710	0.17	0.02 to 1.12	0.065	
	Bichon	1	2036	0.13	0.02 to 0.96	**0.046**	
	Lhasa apso	1	1970	0.13	0.02 to 0.97	**0.046**	
	Golden retriever	0	994	Zero cases	~	~	
	Yorkshire terrier	0	3435	Zero cases	~	~	
Bodyweight relative to breed/sex mean	Lower	160	33,771	Base	–		<0.001
	Equal/Higher	130	16,774	1.50	1.18 to 1.91	**0.001**	
	Unavailable	323	75,878	0.54	0.44 to 0.67	**<0.001**	
Age (years)	<1.0 years	533	61,988	Base	–		<0.001
	1.0 to <2.0 years	80	64,435	0.12	0.09 to 0.15	**<0.001**	
Sex‐neuter	Female‐entire	170	35,609	Base	–		<0.001
	Female‐neutered	96	16,456	1.44	1.10 to 1.90	**0.008**	
	Male‐entire	156	36,749	0.87	0.70 to 1.08	0.216	
	Male‐neutered	107	20,708	1.32	1.01 to 1.71	**0.039**	
	Unavailable	84	16,901	1.45	1.07 to 1.96	**0.016**	

CI Confidence interval

P‐values under 0.05 are shown in bold

n=126,423

As described in the methods, two variables (*purebred* and *adult bodyweight*) were individually added to the final breed‐multivariable model, replacing the breed variable. Purebred dogs had 1.50 times the odds (95% CI 1.24 to 1.83, P=0.001) compared with crossbred dogs. Dogs weighing 10.0 to 19.9 kg (OR: 3.55, 95% CI 2.51 to 5.03, P<0.001) and 20.0 to 29.9 kg (OR: 5.42, 95% CI 3.80 to 7.73, P<0.001) had the highest odds of demodicosis compared with dogs weighing <10.0 kg (Table 3).

**Table 3:** Results for variables introduced individually to replace breed in the final mixed‐effects breed‐multivariable logistic regression model for risk factors associated with diagnosis of demodicosis in dogs aged <2 years under primary veterinary care in the UK

Variable	Category	Cases	Total	Odds ratio	95% CI	Category P‐value	Variable P‐value
Purebred status	Crossbred	132	35,592	Base			<0.001
	Purebred	481	90,339	1.50	1.24 to 1.83	**<0.001**	
Adult bodyweight (>18 months) (kg)	<10.0	45	20,031	Base			<0.001
	10.0 to 19.9	111	13,971	3.55	2.51 to 5.03	**<0.001**	
	20.0 to 29.9	100	8653	5.42	3.80 to 7.73	**<0.001**	
	30.0 to 39.9	21	5401	1.80	1.07 to 3.03	**0.027**	
	≥ 40.0	13	2526	2.50	1.34 to 4.67	**0.004**	
	Unavailable	323	75,841	1.41	1.02 to 1.95	**0.039**	

CI Confidence interval

P‐values under 0.05 are shown in bold

### Risk factors: Dogs over 4 years old

Three risk factors had univariable association with demodicosis at P<0.05: *breed, purebred* and *adult bodyweight*. No association with demodicosis in dogs aged >4 years was detected for s*ex‐neuter, age* or *bodyweight relative to breed/sex mean*. As discussed above, each of the three variables with liberal univariable associations were considered alternative descriptors of a common underlying breed‐based concept and therefore could not be meaningfully co‐entered into multivariable modelling. For this reason, univariable logistic regression with the clinic attended included as a random effect are reported. The univariable random effects model with clinic entered as a random effect was a better model of the data that the non‐random effects model for *breed* P=0.001*, purebred* P=0.002 and *adult bodyweight* P=0.002. The frequencies of missing data for the 228,801 dogs aged >4 years were: *breed* n=664; 0.3%, *purebred* n=664; 0.3% and *adult bodyweight* n=26,037; 11.4%.

Six breeds showed increased odds of demodicosis compared with crossbred dogs: Chinese shar‐pei (OR: 21.91, 95% CI 7.16 to 67.00, P<0.001), shih‐tzu (OR: 15.42, 95% CI 7.91 to 30.06, P<0.001), West Highland white terrier (OR: 8.34, 95% CI 4.18 to 16.65, P<0.001), pug (OR: 5.42, 95% CI 1.23 to 23.90, P=0.026), boxer (OR: 3.90, 95% CI 1.28 to 11.86, P=0.017) and Border terrier (OR: 3.72, 95% CI 1.07 to 12.97, P=0.039). There were four breeds with zero demodicosis cases identified: French bulldog, Border collie, Chihuahua and bichon frise. Purebred dogs had 2.16 times the odds (95% CI 1.23 to 3.77, P=0.007) compared with crossbred dogs. Dogs weighing 20.0 to 29.9 kg (OR: 0.37, 95% CI 0.20 to 0.68, P=0.002) and 30.0 to 39.9 kg (OR: 0.40, 95% CI 0.20 to 0.80, P=0.009) had lower odds of demodicosis compared with dogs weighing <10.0 kg (Table 4).

**Table 4:** Final mixed‐effects univariable logistic regression results for risk factors associated with diagnosis of demodicosis in dogs aged >4 years under primary veterinary care in the UK

Variable	Category	Cases	Total	Odds ratio	95% CI	Category P‐value	Variable P‐value
Breed	Crossbreed	14	51,888	Base			<0.001
	Chinese shar‐pei	4	693	21.91	7.16 to 67.00	**<0.001**	
	Shih Tzu	23	5584	15.42	7.91 to 30.06	**<0.001**	
	West Highland White terrier	19	8504	8.34	4.18 to 16.65	**<0.001**	
	Dogue de Bordeaux	1	590	6.59	0.86 to 50.34	0.069	
	Pug	2	1364	5.42	1.23 to 23.90	**0.026**	
	Boxer	4	3798	3.90	1.28 to 11.86	**0.017**	
	Border terrier	3	3072	3.72	1.07 to 12.97	**0.039**	
	British bulldog	1	1150	3.29	0.43 to 25.09	0.251	
	Golden retriever	3	3755	2.88	0.83 to 10.06	0.097	
	German Shepherd Dog	5	6971	2.66	0.96 to 7.38	0.061	
	Rottweiler	2	2918	2.55	0.58 to 11.25	0.215	
	Others	16	44,373	1.31	0.64 to 2.68	0.465	
	Yorkshire terrier	3	8770	1.27	0.36 to 4.41	0.710	
	Cavalier King Charles spaniel	2	5878	1.25	0.28 to 5.49	0.770	
	Lhasa apso	1	3223	1.19	0.16 to 9.08	0.866	
	Staffordshire bull terrier	5	16,311	1.15	0.41 to 3.21	0.785	
	Springer spaniel	1	3445	1.15	0.15 to 8.78	0.891	
	Cocker spaniel	2	8055	0.90	0.21 to 3.98	0.893	
	Labrador retriever	4	19,095	0.77	0.25 to 2.34	0.643	
	Jack Russell terrier	2	16,210	0.46	0.10 to 2.01	0.299	
	French bulldog	0	295	Zero cases	~	~	
	Border collie	0	7195	Zero cases	~	~	
	Chihuahua	0	2839	Zero cases	~	~	
	Bichon	0	2825	Zero cases	~	~	
Purebred status	Crossbred	14	51,888	Base			0.007
	Purebred	103	176,249	2.16	1.23 to 3.77	**0.007**	
Adult bodyweight (>18 months) (kg)	<10.0	42	52,510	Base			0.012
	10.0 to 19.9	28	56,566	0.62	0.38 to 1.00	0.051	
	20.0 to 29.9	13	44,580	0.37	0.20 to 0.68	**0.002**	
	30.0 to 39.9	10	31,484	0.40	0.20 to 0.80	**0.009**	
	≥ 40.0	11	17,624	0.79	0.40 to 1.53	0.478	
	Unavailable	13	26,037	0.60	0.31 to 1.11	0.101	

CI Confidence interval

P‐values under 0.05 are shown in bold

Comparing results for dogs aged <2 years and dogs aged >4 years, three breeds showed evidence of increased odds of demodicosis in both age groups: Chinese shar‐pei, pug and boxer (Table 5).

**Table 5:** Summary of breeds with increased odds of diagnosis of demodicosis in dogs aged <2 years and in dogs aged >4 years under primary veterinary care in the UK (based on Tables 2 and 4)

	<2 years of age			>4 years of age	
Breed	Odds ratio	95% CI*	Breed	Odds ratio	95% CI*
British bulldog	11	8 to 16	*Chinese shar‐pei*	22	7 to 67
Staffordshire bull terrier	7	6 to 9	Shih tzu	15	8 to 30
*Chinese Shar‐Pei*	7	7 to 12	West Highland white terrier	9	4 to 17
Dogue de Bordeaux	6	3 to 11	*Pug*	5	1 to 24
*Pug*	5	4 to 8	*Boxer*	4	1 to 12
French bulldog	5	3 to 8	Border terrier	4	1 to 13
*Boxer*	2	1 to 4			

CI Confidence interval

Breeds with increased odds in both age groups are in italics

## DISCUSSION

This is the first study to explore the wider presentation of demodicosis in dogs of all ages under primary veterinary care in the UK. Previous studies mainly relied on smaller datasets from teaching hospital or referral practice populations (Sischo *et al*. 1989, Scott & Paradis 1990, Lemarié *et al*. 1996, Bowden *et al*. 2018), reported on populations with demographics and healthcare expected to differ significantly the UK (Nayak *et al*. 1997, Rodriguez‐Vivas *et al*. 2003, Chee *et al*. 2008), or examined only the juvenile form of demodicosis (Bowden *et al*. 2018). This, together with methodological differences relating to study size and case inclusion criteria, may explain the higher prevalence estimates of demodicosis previously reported (0.4 to 23%) compared to the relatively lower overall, 1‐year period prevalence of 0.17% documented here.

In the current study, demodicosis diagnosis was accepted based upon the clinical opinion of the primary‐care clinician and the study did not require evidence of elevated *Demodex* mite populations (Saridomichelakis *et al*. 2007, Fondati *et al*. 2010, Mueller *et al*. 2012). The study data did not include information on the results of skin sampling used for diagnosis. Clinical lesions of demodicosis alone are not pathognomonic and pathology may be masked by secondary infection (Miller *et al*. 2013). Given that full diagnostic testing may not have been carried out in all suspected cases or in all affected dogs where demodicosis was not the suspected cause, it is likely that current study results underestimate the true disease prevalence.

Two demodicosis presentations are recognised based on the extent of the lesions and prognosis: a localised form characterised by a benign clinical course with just a few skin lesions that usually resolve spontaneously, and a generalised form with widespread lesions that do not usually resolve without acaricidal treatment (Miller *et al*. 2013). While the extent of skin lesions may be of prognostic relevance (Miller *et al*. 2013), there are no universally accepted or standard definitions of localised and generalised forms in terms of lesion number, size and distribution. Clinical presentations may fall between those categories and may evolve over time from one to the other. Furthermore, associations between the extent of the disease with either the prognosis or the need for treatment are not absolute (Miller *et al*. 2013). This study did not attempt to distinguish between localised and generalised forms of the disease because the clinical records did not consistently contain sufficiently detailed information to allow this distinction.

Two age‐related presentations of demodicosis are also recognised: “juvenile‐onset” demodicosis, usually first manifesting in puppyhood or young adulthood (3 to 18 months), and adult‐onset demodicosis, usually first manifesting after 4 years of age (Miller *et al*. 2013). Some dogs are first diagnosed between these juvenile and adult periods but fuller examination can often trace these intermediate‐age cases to a younger age, suggesting that these are truly juvenile‐onset cases with a chronic course (Miller *et al*. 2013, Bowden *et al*. 2018). The literature suggests that the majority of demodicosis cases in dogs are juvenile‐onset (Miller *et al*. 2013). The age distribution of demodicosis cases in the current study concurs, with 79.6% of first diagnoses relating to cases under 1.5 years of age. The only other large‐scale study investigating both juvenile and adult‐onset demodicosis reported 56% of diagnoses relating to dogs under 1.5 years in a teaching hospital population with a specialist dermatology service (Bowden *et al*. 2018), which is likely to be biased towards chronic, less typical or difficult‐to‐manage cases.

The expression of juvenile‐onset disease may be linked to the gradual expansion of mite populations (Forton 2011, Ravera *et al*. 2013) following mite transmission in the first days of life (Greve & Gaafar 1966). The genetic, immunological, endocrine or other factors that lead to higher than expected populations of mites associated with skin lesions, focally or more widespread, are poorly understood (Ferrer *et al*. 2014). Less commonly, first manifestation of demodicosis is recognised in adult dogs, and it has been proposed that these dogs first diagnosed when 4 years of age or older are likely to have a truly ‘adult‐onset’ form of the disease (Miller *et al*. 2013). This differentiation matters because juvenile‐onset of the disease is generally attributed to a genetic makeup that confers a mite‐specific immune dysfunction because it usually occurs in the absence of other identifiable immune‐dysregulating factors, whereas true adult‐onset disease carries a high index of suspicion of acquired immune compromise through disease or medication, reducing the host's ability to control mite numbers later in life (Ferrer *et al*. 2014, Miller *et al*. 2013). In these cases, the prognosis depends on the identification and management of relevant co‐morbidity as well as managing the demodicosis (Mueller *et al*. 2012). The manifestation of demodicosis in later adulthood canthus be assumed to depend on the presence of risk factors directly relating to mite control (either overlapping with or distinct from those present injuvenile‐onset disease), plus the presence of co‐risk factors associated with co‐morbidity or age‐related physiological change, even if the condition is not yet clinically apparent (Miller *et al*. 2013).

Taking the perspective that science based on large data resources should be concept‐driven rather than data‐driven (Kell & Oliver 2004), we carried out risk factor analysis separately for two age‐defined phenotypes, that is, for young dogs under 2 years of age to capture cases with “juvenile” onset but allowing for slightly delayed diagnosis, and for dogs over 4 years of age that are deemed to have a high likelihood of the “adult‐onset” course of the disease. A similar approach was taken in a study based on US teaching hospital data (Bowden *et al*. 2018). This approach also takes account of changing breed popularity over time, as has been observed for pure‐bred dogs in the UK (Hanson *et al*. 2000, The Kennel Club 2019) and documented specifically for the pug (O'Neill *et al*. 2016) and the French bulldog (O'Neill *et al*. 2018), by comparing risk within similarly restricted age groups.

The 0.48% 1‐year period prevalence of demodicosis in dogs under 2 years of age documented here is close to the 0.58% prevalence previously reported for juvenile demodicosis in a large study based on general veterinary hospital records in the USA (Plant *et al*. 2011). This may suggest similar demographics in both countries. In line with clinical observation that describes adult‐onset demodicosis as less common (Miller *et al*. 2013), the current study reported 1‐year period prevalence in dogs over 4 years of age (0.05%) as much lower than in young dogs (0.48%). This reflects the observation, recently confirmed (Bowden *et al*. 2018), that many dogs with juvenile‐onset demodicosis are not subsequently diseased in adulthood, and suggests that the poorly understood, mite‐specific immunocompromise suspected in young dogs may be transient or dependent on cofactors present only at this life stage (Mueller *et al*. 2012, Ferrer *et al*. 2014).

Breeds with the highest demodicosis 1‐year period prevalence overall included the bulldog and dogue de Bordeaux. These breeds belong to the same genetic clade which also includes bull terrier breeds (Parker *et al*. 2017), and which forms the genetic basis for pit‐bull types (Olson *et al*. 2015). When looking just at dogs aged under 2 years, five (British bulldog, Staffordshire bull terrier, dogue de Bordeaux, French bulldog and boxer) of the seven breeds with increased odds of demodicosis belonged to this clade. This is in line with a US study which showed that seven of the nine breeds with the highest odds for juvenile demodicosis were breeds of the same genetic clade (Plant *et al*. 2011). Juvenile demodicosis has long been suspected to have a hereditary basis, although published evidence for this is sparse (Ferrer *et al*. 2014). Given the common ancestry of breeds having increased odds of demodicosis at a young age reported in these two independent, large studies, it is reasonable to suspect a significant, shared genetic basis for this disease phenotype. Of the six breeds with increased odds for a diagnosis of demodicosis aged over 4 years, five represent distinct genetic clades; the West Highland white and Border terrier belong to the same clade but are genetically distant within it (Parker *et al*. 2017), and that the bull‐type breeds, some represented in high numbers within the study sample in both age groups and collectively with the highest odds for the juvenile form of the disease, are not represented in the group of dogs aged 4 years and older. This difference in breed spectrum would support the notion that the juvenile and adult‐onset forms of the disease indeed represent different disease phenotypes in terms of aetiological factors (Scott *et al*. 2001).

In the current study, the pug and shar‐pei had high odds for demodicosis in both dogs under 2 years (in line with similar US results (Plant *et al*. 2011)), but also in the group of dogs aged over 4 years. These two breeds do not share a genetic clade with each other or with the bull types (Parker *et al*. 2017). It could therefore be argued that their recurring high presentation among juvenile‐onset and adult‐onset caseloads in combination with high predisposition may reflect the later cases truly being recognised at an older age of chronic disease of juvenile‐onset (Scott *et al*. 2001). A tendency towards a chronic course of the disease, as opposed to a predisposition to truly adult‐onset form cannot be discounted; in both scenarios it is reasonable to suspect that the aetiopathogenesis of demodicosis in these breeds is distinct from, or overlaps with, those of the bull types, assuming that dogs receive similar veterinary care irrespective of breed status.

Three breeds, shih‐tzu, West Highland white terrier and Border terrier, had increased odds only for dogs aged >4 years. West Highland white terrier and shih‐tzu were previously reported as the most frequently affected purebred dogs among a population of dogs with adult‐onset demodicosis in a teaching hospital (Bowden *et al*. 2018). In our study, the odds of demodicosis for shih‐tzu aged under 2 years was 0.44 (95% CI 0.22 to 0.87) times that of crossbreds, whereas the odds of demodicosis for shih‐tzu aged over 4 years was 15.42 (95% CI 7.91 to 30.06) times that of crossbreds. This suggests an aetiopathogenesis distinct from that in bull‐type dogs that generally manifest demodicosis only early in life. A wide range of immunocompromising factors has been proposed as triggers for adult‐onset demodicosis (Scott *et al*. 2001), many of which typically affect older dogs. Based on current observations and the limited understanding of the pathogenesis of canine demodicosis (Ferrer *et al*. 2014), increased odds for disease in dogs aged >4 years may thus reflect a genetic background that promotes both longevity and immunosuppressive cofactors (*i.e*. diseases that receive immunocompromising treatment or immunocompromising disease themselves) and/or an acquired mite‐specific immune dysfunction.

It is difficult to explain why bodyweight above that of the breed/sex mean in dogs under 2 years of age was associated with increased (×1.5) odds for demodicosis. Heavier bodyweight could reflect either increased body condition (*e.g*. obesity) or simply larger inherent stature/size. The current study did not have access to accompanying morphometric data (*e.g*. body condition) on individual dogs and therefore it was not possible to interpret the causes of differing bodyweights (Anderson *et al*. 2018). In humans, obesity is linked to pro‐inflammatory states and increased risk of various inflammatory diseases, including rosacea (Li *et al*. 2017), an inflammatory skin disease associated with high populations of *Demodex* mites (Forton 2011). However, understanding of the negative effects of obesity on canine health is in its infancy (Loftus & Wakshlag 2015), and it may be premature to conclude these parallels, particularly as increased bodyweight was not also evident as a risk factor in adult‐onset demodicosis. The association between juvenile‐onset demodicosis and neutered status may reflect reverse causality whereby demodicosis cases may have increased probability of neutering after diagnosis (Jacka *et al*. 2015).

The study had some limitations. Primary‐care clinical data are not recorded primarily for research purposes and thus were limited by some missing data as well as reliance on accurate and thorough record‐keeping of the clinicians. As discussed above, the study relied on diagnoses recorded by primary‐care clinician teams. Varying diagnostic criteria across clinics and individual veterinarians may have introduced some selection bias to the final results that is difficult to quantify, For uncommon breeds, the low numbers of both cases and dogs overall result in wide CIs for the prevalence and odds ratio values. This means that such breed association findings should be treated with caution. The study clinics were a convenience sample and may not be fully representative of the overall veterinary practice structure and caseloads in in the UK. Cognitive biases whereby belief that certain age groups, breeds or animal types are predisposed to demodicosis may have increased the diagnostic probability in such animals. These so‐called cognitive dispositions to respond can affect other aspects of clinical activity such as treatment and prognosis as well as diagnosis but their impact may be reduced by strategies including reflection on problem solving and access to current relevant clinical evidence (Croskerry 2003).

## CONCLUSIONS

This study of the general dog population under primary veterinary care showed 10 times higher one‐year period prevalence of demodicosis in dogs aged under 2 years (0.48%) than in dogs aged over 4 years (0.05%). It identified breed as a major risk factor for both age groups. Differing spectra of predisposed breeds across the juvenile‐onset and adult‐onset forms of the disease, and in particular the dominance of bull type breeds in clade W from the 23 identified genetic clades (Parker *et al*. 2017) in juvenile‐onset demodicosis, supports the concept of distinct age‐related aetiopathogenetic phenotypes of this complex disease. These findings will help inform case selection for future work investigating primary genetic risk factors, and also relevant cofactors for disease expression. This is particularly relevant information for veterinary surgeons given the marked rise in popularity in recent years of breeds such French bulldog, pug, British bulldog and Staffordshire bull terrier with increased odds of juvenile demodicosis in the UK.

### Authors' contributions

All authors made substantial contributions to conception and design, acquisition and extraction of data, and to analysis and interpretation of the results. All authors were involved in drafting and revising the manuscript and gave final approval of the version to be published. Each author agrees to be accountable for all aspects of the accuracy or integrity of the work.

### Conflict of interest

The authors have no conflicts of interest to declare.

## Data Availability

The datasets generated and analysed during the current study are publicly available on the RVC data repository.
